# Genome-wide analyses reveal lineage specific contributions of positive selection and recombination to the evolution of *Listeria monocytogenes*

**DOI:** 10.1186/1471-2148-8-233

**Published:** 2008-08-12

**Authors:** Renato H Orsi, Qi Sun, Martin Wiedmann

**Affiliations:** 1 Department of Food Science, Cornell University, Ithaca, NY, USA; 2 Computational Biology Service Unit, Cornell University, Ithaca, NY, USA

## Abstract

**Background:**

The genus *Listeria *includes two closely related pathogenic and non-pathogenic species, *L. monocytogenes *and *L. innocua*. *L. monocytogenes *is an opportunistic human foodborne and animal pathogen that includes two common lineages. While lineage I is more commonly found among human listeriosis cases, lineage II appears to be overrepresented among isolates from foods and environmental sources. This study used the genome sequences for one *L. innocua *strain and four *L. monocytogenes *strains representing lineages I and II, to characterize the contributions of positive selection and recombination to the evolution of the *L. innocua*/*L. monocytogenes *core genome.

**Results:**

Among the 2267 genes in the *L. monocytogenes/L. innocua *core genome, 1097 genes showed evidence for recombination and 36 genes showed evidence for positive selection. Positive selection was strongly associated with recombination. Specifically, 29 of the 36 genes under positive selection also showed evidence for recombination. Recombination was more common among isolates in lineage II than lineage I; this trend was confirmed by sequencing five genes in a larger isolate set. Positive selection was more abundant in the ancestral branch of lineage II (20 genes) as compared to the ancestral branch of lineage I (9 genes). Additional genes under positive selection were identified in the branch separating the two species; for this branch, genes in the role category "Cell wall and membrane biogenesis" were significantly more likely to have evidence for positive selection. Positive selection of three genes was confirmed in a larger isolate set, which also revealed occurrence of multiple premature stop codons in one positively selected gene involved in flagellar motility (*flaR*).

**Conclusion:**

While recombination and positive selection both contribute to evolution of *L. monocytogenes*, the relative contributions of these evolutionary forces seem to differ by *L. monocytogenes *lineages and appear to be more important in the evolution of lineage II, which seems to be found in a broader range of environments, as compared to the apparently more host adapted lineage I. Diversification of cell wall and membrane biogenesis and motility-related genes may play a particularly important role in the evolution of *L. monocytogenes*.

## Background

Positive selection and recombination are two evolutionary forces that are clearly important in the evolution of many microorganisms [1-9]. A number of studies of natural bacterial populations have found evidence for positive selection in specific genes, including in *Escherichia coli *[10], *Neisseria meningitides *[1,11], and *Listeria monocytogenes *[4,9,12]. Recent whole-genome analyses of *E. coli *[6,8] and *Streptococcus *[2] have also confirmed the importance of positive selection during evolution of these pathogens. One study specifically suggested that, in bacteria, up to 2 × 10^-5 ^mutations per genome, per generation, are beneficial [5] and another study reported that more than half of the amino acid substitutions between *E. coli *and *Salmonella enterica *appear to have been fixed by positive selection [3]. Furthermore, gains in fitness associated with nonsynonymous changes have also been confirmed in *in vitro *experiments [13,14]. Lateral gene transfer (LGT), followed by incorporation of homologous DNA into the genome, appears to be common in many bacteria and occurrence of homologous recombination has been described in many microorganisms [1,2,4,9,11,15,16]. Bacterial populations can differ considerably in frequency of recombination though; while some populations appear to be panmictic (e.g., *Helicobacter pylori *[17]), others seem to show much more limited recombination (e.g., *Borrelia burgdorferi *[18]).

In absence of recombination, positive selection can be inefficient due to clonal interference and/or genetic load. In the case of "clonal interference", advantageous mutations that arise in different lineages of the same population compete against each other for fixation, which can slow down the fixation of advantageous mutations, and can result in loss of advantageous mutations. "Genetic load" refers to the increase in frequency or fixation in the population of disadvantageous mutations that are linked to advantageous mutations. Recombination not only allows advantageous mutations present in different lineages to be combined and fixed in the same lineage, thus preventing clonal interference [19-23], but also can break the linkage between the advantageous and disadvantageous mutations, thus counteracting "genetic load" [24-27]. Positive selection may also play an important role in facilitating maintenance of fragments introduced by recombination in a given population if these fragments confer a selective advantage to the recipient organism.

The genus *Listeria *includes both mammalian pathogenic species (i.e., *L. monocytogenes*, a human and animal pathogen and *L. ivanovii*, an animal pathogen) as well as non-pathogenic species (e.g., *L. innocua, L. welshimeri*) [28]. *L. monocytogenes *is a facultative intracellular foodborne pathogen, which can cause severe invasive human disease with case mortality rates of 20% [29]. Adaptive immunity against *L. monocytogenes *is believed to be mainly cellular-mediated [30], although natural antibodies also seem to play a role in protection [31,32]. *L. monocytogenes *also has the ability to grow under a wide range of environmental stress conditions, including temperatures ranging from 0°C to 45°C [33,34], pH ranging from 4 to 9.6 [35,36] and salt concentration of up to 10% [37], facilitating its foodborne transmission. *L. monocytogenes *isolates form a structured population with at least four phylogenetic lineages, including lineages I and II, which are common and lineages IIIA/C and IIIB, which are rare [12,38]. Although isolates from all four lineages have been associated with human listeriosis, most human listeriosis cases and outbreaks have been associated with lineage I isolates, in particular those of serotype 4b [39,40]. Lineage II isolates, on the other hand, seem to be overrepresented among isolates from foods and environmental sources, and underrepresented among human clinical cases [41,42]. These findings suggest that lineage I isolates are more virulent than lineage II isolates, which has been supported by a risk assessment [43] as well as by observations that lineage I isolates, on average, show higher measures of tissue culture pathogenicity as compared to lineage II isolates [42,44,45]. In addition, a considerable proportion of lineage II isolates, but only few lineage I isolates, are virulence-attenuated due to nonsense and frameshift mutations in virulence genes resulting in truncated proteins [9,46,47]. Combined, these observations have led to the conclusions that *L. monocytogenes *lineage I may be host-adapted, while lineage II may represent an environmentally-adapted group [41].

Interestingly, the pathogenic *L. monocytogenes *is most closely related to the non-pathogenic *L. innocua*. Consequently, the *L. innocua/L. monocytogenes *lineage within the genus *Listeria *has been used as a model system to study the evolution of pathogenicity characteristics, including through comparative genome analyses [48,49]. While gene presence/absence patterns in these two sister species have been probed through both genome sequencing [49] and macroarray [50] studies, facilitating identification of confirmed and putative virulence genes, evolutionary patterns of the core genome of the *L. innocua/L. monocytogenes *lineage have not yet been comprehensively studied. We used genome sequences available for *L. innocua *[49] as well as for two *L. monocytogenes *lineage I and two lineage II strains [49,51] to investigate the contributions of recombination and positive selection to the evolution of the core genome in these *Listeria *lineages and to gain a better understanding of mechanism that may be important in the evolution of core genome genes during diversification of bacterial pathogens.

## Methods

### Genome data

Full genome sequence data for four *L. monocytogenes *isolates and one *L. innocua *isolate were used for this study (Table 1). Protein and gene sequence data for these five isolates were retrieved from the Comprehensive Microbial Resource [52]. The *L. monocytogenes *isolates represented two serotype 4b lineage I strains (F2365, H7858) as well as two serotype 1/2a lineage II strains (EGD-e, F6854). While F2365 shows at least 20 authentic mutations resulting in premature stop codons and demonstrates some atypical invasion characteristics [53], the genome for this strain was included in our analyses to provide us with increased power for our analyses and an appropriate number of sequences to perform the lineage specific analyses for positive selection. Genes with premature stop codons were excluded from the analyses for positive selection and recombination; presence of these genes in F2365 thus did not affect our analyses. To identify orthologous genes found in all five genomes (i.e., genes representing members of the *L. monocytogenes*/*L. innocua *lineage core genome), the predicted protein sequences of each gene from each genome were clustered using BLAST and TribeMCL [54]. Gene clusters were initially identified using TribeMCL (run with the inflation value set at 2) using BLAST cutoff values of 1e-150, followed by identification of clusters containing less conserved genes (using BLAST cutoff values of 1e-100, 1e-50, and 1e-30). This stepwise approach was used to minimize inclusion of multiple genes from the same genome in a given cluster; the majority of the clusters identified had no more than one gene from each genome. Four clusters contained four sequences from each EGD-e, CLIP 11262, F2365, and H7858 as well as two identical or nearly sequences from F6854 (these four sequences were considered paralogs in F6854); only the F6854 sequence that matched the length of the other genes in this cluster was retained. Only clusters containing five sequences, one from each genome, were further analyzed.

**Table 1:** Strains and genomes analyzed.

Strain	Serotype	Lineage	Species	Genome size (nt)	No. of CDS^(1)^	Ref.
EGD-e	1/2a	II	*L. monocytogenes*	2,944,528	2846	[49]
F6854	1/2a	II	*L. monocytogenes*	2,953,211	2945	[51]
F2365	4b	I	*L. monocytogenes*	2,905,310	2821	[51]
H7858	4b	I	*L. monocytogenes*	2,893,921	3007	[51]
CLIP 11262	6a	-	*L. innocua*	3,011,209	2968	[49]

^(1) ^number of coding sequences used for cluster analysis.

Orthologs grouped in the same clusters were aligned using the Clustal W method [55]. Alignments were scanned for frameshift mutations, presence of stop codons, and gene sequences with unequal length; in addition, number of informative sites, average nucleotide diversity (π), overall identity, and identity in the first and last 15 nucleotides were obtained for each cluster. Alignments identified as having low identities or containing sequences with different lengths were manually evaluated using the program BioEdit [56] and trimmed or otherwise edited if necessary. If alignments contained frameshift mutations generated by indels (insertion/deletion) followed by another indel that restored the original frame, the alignment was edited by removing the region between the frameshift mutations. Final alignments were used for positive selection and recombination analyses as detailed below.

Each gene cluster was also assigned to one of 19 COGs (Clusters of Orthologous Groups of proteins) or the category "not in COG" based on the EGD-e genome annotation available in the NCBI genome database. The effective number of codons used in a gene (Nc), a measure of the codon bias, was assessed using the program "chips" implemented in the EMBOSS package [57]. Nucleotide diversity and number of informative sites were obtained from PhiPack outputs (see below under "Recombination analyses"). Genes also were classified as encoding (i) cell wall proteins, (ii) secreted proteins or (iii) membrane proteins based on the classifications listed in the LEGER database [58].

### Positive selection analysis

**Figure 1:**
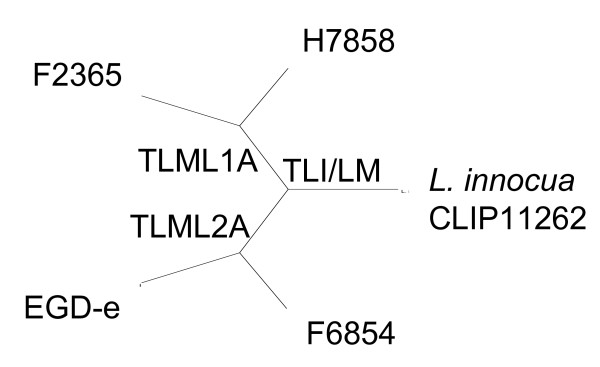
**Unrooted phylogenetic tree of the five strains used in the genome-wide analyses.** This tree represents the consensus tree of 2267 gene cluster alignments used for analyses. Branches tested for positive selection are indicated as TLM1A (Test *L. monocytogenes *lineage IAncestral), TLM2A (Test *L. monocytogenes *lineage IIAncestral), and TLI/LM (Test *L. innocua*/*L. monocytogenes*).

Genes under positive selection were identified using codeml as implemented in PAML version 3.15 [59]. The models implemented in PAML allow for identification of genes under positive selection (as well as specific sites that are under positive selection in a gene) even if the overall d_N_/d_S _ratio (ω) for a gene is < 1. We employed two types of tests implemented in PAML to identify genes under positive selection. An overall test for positive selection (Test Overall; TO) was carried out using the null model M1a (Nearly-neutral) and the alternative model M2a (Positive selection) [60]; this test identifies genes under positive selection in any or all of the branches of a given phylogeny. To identify genes that are under positive selection in specific branches of the *L. monocytogenes/L. innocua *phylogeny, the branch-site test2 described by Zhang et al. [61] was used. This test was used to identify genes under positive selection in three branches (Fig. 1), including (i) the ancestral branch of *L. monocytogenes *lineage I (Test *L. monocytogenes *lineage IAncestral; TLM1A), (ii) the ancestral branch of *L. monocytogenes *lineage II (Test *L. monocytogenes *lineage IIAncestral; TLM2A), and (iii) the branch separating *L. monocytogenes *and *L. innocua *(Test *L. innocua*/*L. monocytogenes*; TLI/LM). Because the sequence of the *L. innocua *and *L. monocytogenes *ancestor is unknown, TLI/LM cannot differentiate between positive selection in *L. innocua *and positive selection in the ancestor of *L. monocytogenes*. No test was performed to test for evidence of positive selection among genes within a given lineage. Initially, one universal phylogenetic tree (Fig. 1), representing the consensus tree of the 2267 genes analyzed, was used for all PAML analyses. For all genes that were identified as being under positive selection, gene-specific trees were constructed and TO and branch-specific PAML analyses were re-run if the gene-specific tree differed from the consensus tree. For eight genes, gene-specific trees differed from the universal tree. PAML analysis with gene-specific trees confirmed positive selection for five genes, while for three genes analyses with the gene-specific trees did not find any evidence for positive selection (these genes were thus not considered to be under positive selection).

For each test, nested models (one null model that does not allow for positive selection and one alternative model that allows for positive selection) were compared using a Likelihood Ratio Test (LRT) as described by Yang et al. [62]. For each model, three replicates were generated and the maximum likelihood values for each model were used in the LRT. Genes with negative LRT values were re-run 10 times and the maximum values for each model were used for LRT. Persistent negative LRT values were rounded to zero (*P *= 1). For all branch-specific tests, one degree of freedom was used to calculate *p*-values, while for the overall test, two degrees of freedom were used to calculate *p*-values.

### Recombination analyses

Four tests were used initially to assess gene clusters for evidence of intragenic recombination, including Sawyer's test implemented in GENECONV version 1.81 [63] as well as Neighbor Similarity Score (NSS), Maximum χ^2^, and the Pairwise Homoplasy Index (PHI), the last three implemented in PhiPack [64]. For GENECONV analyses, the parameter g-scale was set to 1; this setting allows polymorphisms within the recombinant fragment, increasing the likelihood of the test to identify ancient recombination events or events where the donated recombinant fragment is similar, but not identical to the recombinant sequence in the alignment. In the GENECONV analyses, only inner fragments were considered. For Maximum χ^2^, a fixed window size of 2/3 the number of polymorphic sites was used. For PHI, a window size of 50 nucleotides was used. *p*-values were estimated using 10,000 permutations of the alignment for GENECONV and 1,000 permutations for NSS, Maximum χ^2 ^and PHI. Therefore, for all recombination tests, the *p*-values represent the proportion of test statistics of the permuted alignments that were at least as extreme as the observed test statistic.

ClonalFrame version 1.1 [65] was used on selected genes to estimate recombination breakpoints and to help identify the most likely recipients in a given recombination event. ClonalFrame assumes that recombination events generate new polymorphisms in the population and is most useful for data sets where the donor of the recombinant fragment is not present in the data set [65]. Nevertheless, the program can also be used to identify recombination between sequences in the data set, although it might underestimate the amount of recombination in alignments where the donor and recipient are closely related [65]. Analyses were based on two independent runs of the program both using the same settings (100,000 burn-in iteration and data collection for an additional 100,000 iterations; default settings were used for all other parameters). A 95% consensus tree was obtained from these two runs, and only those branches present in the 95% consensus tree were analyzed for recombination.

### Statistical analyses

Correction for multiple testing was performed using the procedure reported by Benjamini & Hochberg [66] as implemented in the program Q-Value [67] with the proportion of expected true null hypotheses set to 1 (π_0 _= 1). For each *p*-value, the *q-*value (the expected proportion of false positives among the significant tests) was calculated. Corrections were performed separately for each test (e.g., GENECONV, NSS, etc.; TO, TLI/LM, etc.) to account for testing of multiple genes (i.e., 2237 genes). As the tests used for positive selection are already conservative [61], a false discovery rate (FDR) of 20% was used for the positives selection analyses. For recombination analyses, an FDR of 10% was used to compensate the fact that no correction for multiple tests (Sawyer's test, NSS, Maximum χ^2 ^and PHI) was carried out due to the high correlation among the tests.

Correlation between COGs and positive selection, recombination, and gene parameters (e.g., gene length, codon bias, nt diversity) were carried out using chi-square tests, Fisher's exact tests, and U-tests implemented in SAS. For association between genes in a given COG category, COGs that were numerically overrepresented among genes under recombination or positive selection were tested for the significance of associations using one-sided tests; Bonferroni corrections were performed based on the number of one-sided tests performed. Significance was set at 5%.

### Confirmation of positive selection and recombination patterns in selected genes in a larger isolate population

To probe whether the positive selection and recombination patterns determined using genome wide analyses on five isolates were representative for larger populations, a diverse set of 40 additional *L. monocytogenes *isolates (Additional file 1) was assembled and used to determine the sequences for five genes (i.e., *cheA*, *phoP*, *lmo0693*, *flaR *and *lmo2537*) for positive selection and recombination analyses. Isolates were selected to represent the genetic diversity of *L*. *monocytogenes*, including lineage I (19 isolates), II (13 isolates), IIIA/C (5 isolates), and IIIB (3 isolates), as well as diverse sources (e.g., foods, human clinical cases; see Additional file 1). Experiments with biohazardous materials were approved by the Cornell Institutional Biosafety Committee (MUA #15520). Nucleotide sequences for these five genes have been deposited in GenBank as alignments (PopSet accession numbers 164520363, 164520263, 164520173, 164520083, 164519993).

PCR amplification of the five selected genes was carried out using primers and conditions described in Additional file 2. PCR fragments were purified using Exonuclease I (0.5 U/μl) and Shrimp alkaline phosphatase (0.05 U/μl) (USB, NEB) and sequenced (at the Biotechnology Resource Center, Cornell University) using Big Dye Terminator chemistry and AmpliTaq-FS DNA Polymerase and an automated 3730 DNA Analyzer. Sequences were proofread and aligned using Clustal W using Seqman and Megalign, as implemented in Lasergene 7.2.1. Alignments were used for positive selection and recombination analyses as described above.

### Swarming assays

Swarming assays were performed with six isolates representing each of the six different mutations leading to premature stop codons (i.e., FSL C1-057, FSL F2-649, FSL E1-123, FSL F2-663, FSL S4-766 and FSL F2-086), three isolates bearing full length *flaR *(i.e. 10403S, FSL F2-661 and FSL J1-208), and a non-motile isogenic 10403S Δ*flaA *strain [68], which harbors an in-frame deletion of the gene that encodes the flagellin subunit in *L. monocytogenes*. We furthermore constructed an isogenic in-frame *flaR *null mutant in *L. monocytogenes *10403S background using Splicing-by-Overlap (SOEing) PCR and allelic exchange, as previously described [69] for use in swarming assays.

Swarming abilities of *L. monocytogenes *strains were evaluated on semi-soft agar. Strains were initially grown for 24 h at 37°C on BHI agar and colonies were used to stab-inoculate BHI semi-soft agar (0.4%). Swarming ability was assessed by measuring colony area using SigmaScan Pro 5.0 (SPSS Inc., Chicago, IL) for each strain after incubation at room temperature for 48 h. Swarming area for each mutant strain was normalized to the swarming area for strain 10403S, which was set at 100%.

## Results

### Initial identification and characterization of the *L. monocytogenes/L. innocua *core genome

**Figure 2:**
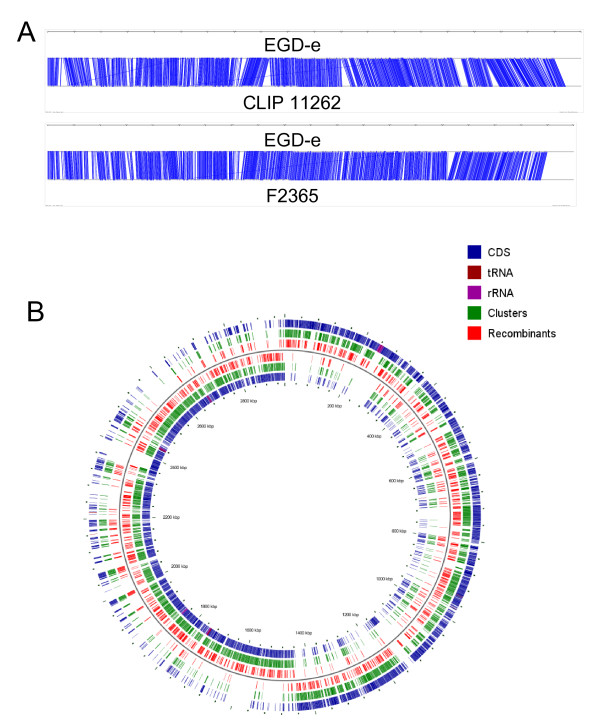
**Schematic representations of the genes analyzed including (A) relative position of the orthologs in EGD-e, CLIP 11262 and F2365; and (B) circular chromosome of EGD-e.** In "B" all protein coding, tRNA, and rRNA genes are shown in blue, brown, and purple; all genes analyzed are shown in green and genes with evidence for recombination (at least one test significant) are shown in red. There was no evidence for spatial clustering of genes with evidence for recombination (*P *= 0.957; U-test).

Using BLAST and TribeMCL (as detailed in the "Methods"), we identified 2267 orthologous genes present in all five genomes, representing an initial definition of the core genome for the *L. monocytogenes/L. innocua *lineage. The orthologs were highly syntenic in the two *L. monocytogenes *lineages and *L. innocua *(Fig. 2A). The 2267 orthologous genes identified represent 76% of the coding genes identified in *L. innocua *CLIP11262 and 80% of the coding genes identified in *L. monocytogenes *EGD-e and F2365 (i.e., the two closed *L. monocytogenes *genomes). Thirty of the 2267 genes in the core genome had ≤ 1 informative site and were thus not used in subsequent analyses; a final set of 2237 genes was thus used in all genome-wide analyses (shown in green in Fig. 2B).

Genes in COGs "Energy production and conversion", "Amino acid transport and metabolism", "Carbohydrate transport and metabolism", "Replication, recombination and repair", "Defense mechanisms", and "Cell wall/membrane biogenesis" showed a significant tendency to be longer than genes in other COGs (Table 2). Although genes categorized into the COGs "Amino acid transport and metabolism", "General functional prediction", "Defense mechanisms", "Coenzyme transport and metabolism", "Replication, recombination and repair", and "Cell wall/membrane biogenesis" showed a significant tendency for a greater number of informative sites than the genes in other COGs (Table 2), only genes in the last three COGs showed a significant tendency for a higher average genetic diversity (π)(Table 2).

**Table 2:** Associations between COGs and descriptive variables.

COG	Number of gene analyzed	Bonferroni-corrected P value for one-sided U-test testing for associations between genes in a given COG and^(1)^				
		
		> length	> nt diversity	> Number of Informative sites	> Codon bias^(2)^	< Codon bias^(2)^
Energy production and conversion	100	< 0.002	ND	NS	0.007	ND
Cell cycle control, mitosis and meiosis	20	NS	ND	ND	NS	ND
Amino acid transport and metabolism	181	< 0.002	NS	< 0.002	NS	ND
Nucleotide transport and metabolism	63	NS	ND	NS	NS	ND
Carbohydrate transport and metabolism	186	< 0.002	ND	NS	NS	ND
Coenzyme transport and metabolism	87	NS	< 0.001	0.004	ND	0.047
Lipid transport and metabolism	51	NS	NS	NS	NS	ND
Translation	91	NS	ND	ND	< 0.001	ND
Transcription	187	ND	ND	ND	ND	< 0.002
Replication, recombination and repair	92	< 0.002	< 0.001	< 0.002	ND	NS
Cell wall/membrane biogenesis	76	< 0.002	< 0.001	< 0.002	NS	ND
Cell motility	40	NS	ND	NS	ND	NS
Posttranslational modification, protein turnover, chaperones	52	ND	ND	ND	0.021	ND
Inorganic ion transport and metabolism	104	NS	ND	NS	ND	NS
Secondary metabolites biosynthesis, transport and catabolism	31	ND	NS	NS	ND	NS
General function prediction only	254	NS	NS	0.008	ND	NS
Function unknown	161	ND	NS	ND	ND	NS
Signal transduction mechanisms	104	NS	ND	NS	ND	< 0.002
Intracellular trafficking and secretion	37	NS	ND	ND	NS	ND
Defense mechanisms	51	< 0.002	NS	0.020	ND	NS
Not in COGs	511	ND	ND	ND	ND	NS

^(1) ^">" or "<" indicates the direction of the one-sided tests (i.e. column "> Codon bias" shows Bonferroni-corrected *p*-values for associations between genes in a given COG and higher codon bias (as compared to the genes in other COGs), while the column "< Codon bias" test shows Bonferroni-corrected *p*-values for genes in COGs with lower codon bias as compared to genes in other COGs; "ND" = Not determined (tests were not performed for COGs that showed values that were not consistent with the tested alternative hypothesis, e.g., if the average gene length for genes in a given COG was below average than we did not test for an association of this COG with increased gene length); "NS". Not significant.

^(2)^Tests for codon bias were performed using Nc values; a lower Nc indicates increased codon bias.

Genes categorized into the COGs "Energy production and conversion", "Translation", and "Posttranslational modification, protein turnover, chaperone" showed a significant tendency for a higher codon bias (Table 2), possibly reflecting their housekeeping roles and higher expression rates [70-72]. Conversely, genes in the COGs "Coenzyme transport and metabolism", "Transcription", and "Signal transduction mechanisms" showed a tendency for lower codon bias (Table 2), possibly because these genes are not constitutively expressed and are not highly expressed in the cell, lowering the constraint for preferential codons usage [70-72].

### A considerable number of *L. monocytogenes *and *L. innocua *genes show evidence for recombination

Among the 2237 orthologs tested, 1097 genes (representing approx. 49% of the genes in the *L. monocytogenes/L. innocua *core genome) showed significant evidence (FDR < 10%) for recombination in at least one of the four recombination tests used (these genes are shown in red in Fig. 2b). GENECONV, NSS, Maximum χ^2 ^and PHI identified 508, 460, 900, and 252 orthologs, respectively, with significant evidence for recombination. A total of 516, 282, 156, and 143 orthologs showed significant evidence for recombination in one, two, three and all four tests, respectively. Genes with evidence for recombination showed a tendency to have longer alignments (*P *< 0.001; One-sided U-Test), lower codon bias (*P *= 0.013), higher nucleotide diversity (*P *< 0.001), and more informative sites (*P *< 0.001) then genes with no evidence for recombination. These findings are consistent with the expectation that, by chance, shorter genes are less likely to be involved in intragenic recombination, but also the observation that shorter sequences provides less power in the analyses for evidence of recombination [64,73].

When genes that encode for (i) cell wall proteins, (ii) secreted proteins or (iii) membrane proteins (based on the listings in LEGER) were tested for their prevalence among genes with evidence for recombination, these three genes classes (i.e., cell wall proteins, secreted proteins, and membrane proteins) were under-represented (*P *= 0.002, *P *= 0.001, and *P *= 0.013, respectively; one-sided Fisher's exact test) among the 1097 genes with significant evidence for recombination, suggesting that these genes are less likely to have experienced recombination.

Genes in three COGs are overrepresented among the genes that show evidence for a history of recombination (Table 3). For example, the "Carbohydrate transport and metabolism" COG was significantly overrepresented among the 1097 genes with evidence for recombination in at least one of the four tests (*P *= 0.012). Genes in this COG were also significantly more likely to have low *p*-values (indicative of significant evidence for recombination) as compared to genes in the other COGs for each of the four tests (as determined by U-tests; see Table 3). For three tests (NSS, Maximum χ^2^, and PHI), genes in the "Amino acid transport and metabolism" COG were significantly more likely to have low *p*-values (indicative of significant evidence for recombination), as compared to genes in the other COGs, respectively (Table 3). These data may indicate that recombination allows *L. monocytogenes *and *L. innocua *to rapidly generate and acquire diversity in genes involved in carbohydrate and amino acid transport and metabolism, which may facilitate adaptation to environments that differ in nutrient availability (e.g., host and non-host associated environments). As these two COGs also showed a tendency to have longer genes, the association between these COGs and recombination could also be due to an increased power to detect recombination.

**Table 3:** Association between COGs and recombination

COG	*P*-values for association with recombination^(1)^	*P*-values for association with recombination test^(2)^			
		
		GENECONV	NSS	**Maximum **χ^2^	PHI
Carbohydrate transport and metabolism	0.012	0.001	< 0.001	0.014	0.032
Amino acid transport and metabolism	NS	NS	0.001	0.026	0.002
Defense mechanisms	NS	NS	0.022	NS	NS

^(1) ^Fisher's exact test with Bonferroni correction; NS = not significant;

^(2) ^U-test with Bonferroni correction.

### Thirty-six *L. monocytogenes *and *L. innocua *genes show evidence for positive selection

PAML identified 36 genes under positive selection (FDR < 20%) with either the overall test (TO) or the branch specific tests, including one gene (*lmo2178*) identified with two branch specific tests and one gene (*lmo0782*) identified with TO and two branch specific tests (Table 4). Three genes were identified as being under positive selection with the overall test (TO). Seven were identified with the *L. innocua *test (TLI/LM), 9 with the *L. monocytogenes *lineage I ancestor test (TLM1A), and 20 with the *L. monocytogenes *lineage II ancestor test (TLM2A). Genes under positive selection showed a tendency to have more informative sites and to be longer than genes not under positive selection (*P *= 0.001 and *P *= 0.031; one-sided U-Test), probably as these two factors may increase the power of the test for positive selection. Positive selection was not associated with nucleotide diversity or codon bias.

**Table 4:** Genes under positive selection.

Gene locus	Gene description (gene symbol)	COG^(1)^	Recombination^(2)^	Branch under pos. selection	Q-value	ω^(3)^	p^(4)^	BEB (*P *> 95%)^(5)^
Lmo0098	PTS system, mannose/fructose/sorbose family, IID component (*mptD*)	NCOG	GCV; MAX	LI/LM	0.170	472.58	0.004	-
Lmo0099	conserved hypothetical protein	NCOG	-	LI/LM	0.170	∞	0.009	-
Lmo0139	conserved hypothetical protein	NCOG	-	LM2A	0.160	56.74	0.054	95
Lmo0297	PRD/PTS system IIA 2 domain protein	K; G; T	GCV; MAX; NSS; PHI	LM2A	0.164	∞	0.012	499
Lmo0397	conserved hypothetical protein	S	GCV; MAX	LM2A	0.1264	∞	0.043	-
Lmo0429	glycosyl hydrolase, family 38	G	GCV; MAX; NSS; PHI	LM2A	0.0325	977.51	0.008	667
Lmo0455	conserved hypothetical protein	T; Q	GCV; MAX; NSS; PHI	LM2A	0.1214	∞	0.004	-
Lmo0653	conserved hypothetical protein	S	MAX	LM2A	0.033	∞	0.012	306
Lmo0658	endonuclease III domain protein	L	MAX; NSS	LM2A	0.108	∞	0.023	209
Lmo0692	chemotaxis protein CheA (*cheA*)	T; N	GCV; MAX; NSS; PHI	LM1A	0.156	∞	0.002	-
Lmo0693	flagellar motor switch domain protein	N; U	-	LM2A	0.007	∞	0.022	-
Lmo0695	conserved hypothetical protein	NCOG	MAX; NSS; PHI	LM1A	0.175	∞	0.014	-
Lmo0732	cell wall surface anchor family protein	NCOG	GCV	LM1A	0.185	6.92	0.076	-
Lmo0782	PTS system, mannose/fructose/sorbose family, IIC component (*mpoD*)	NCOG	GCV; MAX	Overall;	0.146;	88.23;	0.008;	-
				LM1A;	0.052;	∞;	0.0001;	-
				LM2A	0.098	∞	0.004	-
Lmo0785	sigma-54 dependent Kal regulator (*manR*)	K; T	GCV; MAX	LM2A	0.129	1.00	0.000	-
Lmo0872	major facilitator family transporter	G	GCV; MAX; NSS	LM2A	0.137	∞	0.008	-
Lmo0910	putative membrane protein	R	MAX; NSS	LM1A	0.019	∞	0.012	-
Lmo1146	conserved hypothetical protein	NCOG	GCV; MAX	LI/LM	0.046	223.37	0.023	169
Lmo1164	PduO protein (*pduO*)	S; R	GCV	LI/LM	0.170	195.87	0.038	-
Lmo1412	DNA topology modulation protein FlaR (*flaR*)	F	-	LM2A	0.160	8.56	0.139	12; 37; 68
Lmo1424	transporter, NRAMP family (*mntH*)	P	GCV	LM1A	0.185	1.00	0.000	-
Lmo1523	GTP pyrophosphokinase (*relA*)	K; T	-	LM2A	0.033	1.00	0.000	-
Lmo1529	preprotein translocase, YajC subunit	U	-	LI/LM	0.170	∞	0.011	-
Lmo2102	glutamine amidotransferase, SNO family (*pdxT*)	H	GCV; MAX; NSS; PHI	LM1A	0.185	∞	0.017	66
Lmo2121	glycosyl transferase, family 65	G	GCV; MAX; NSS	LM1A	7.6E-06	35.31	0.031	722; 723; 725; 729; 730; 744; 752;
Lmo2178	cell wall surface anchor family protein	M	GCV; MAX; NSS; PHI	LI/LM;	0.193;	15.92;	0.001;	-
				LM2A	0.137	5.34	0.025	1769
Lmo2215	ABC transporter, ATP-binding protein	V	MAX; NSS	Overall	0.188	14.23	0.008	-
Lmo2222	Ser/Thr protein phosphatase family protein	L	GCV; MAX	LM2A	0.160	∞	0.021	253
Lmo2446	glycosyl hydrolase, family 31	G	GCV; MAX; NSS; PHI	LM2A	0.003	∞	0.0001	-
Lmo2596	ribosomal protein S9 (*rpsI*)	NCOG	-	LM2A	0.021	∞	0.010	-
Lmo2611	adenylate kinase (*adk*)	F	GCV; MAX	LM2A	0.120	24.80	0.013	-
Lmo2724	conserved hypothetical protein	S	NSS	LI/LM	0.170	∞	0.008	-
Lmo2802	glucose-inhibited division protein B (*girB*)	M	GCV; MAX; NSS; PHI	LM1A	0.046	∞	0.013	-
Lmo2804	conserved hypothetical protein	NCOG	GCV; MAX; NSS; PHI	Overall	6.5E-17	16.53	0.044	-
Lmo2824	D-isomer specific 2- hydroxyacid dehydrogenase family protein	E; H	GCV; MAX; NSS	LM2A	0.160	229.62	0.003	-

^(1) ^NCOG: Not in COGs; K: Transcription; G: Carbohydrate transport and metabolism; T: Signal transduction mechanisms; S: Function unknown; Q: Secondary metabolites biosynthesis, transport and catabolism; L: Replication, recombination and repair; N: Cell motility; U: Intracellular trafficking and secretion; R: General function prediction only; F: Nucleotide transport and metabolism; P: Inorganic ion transport and metabolism; E: Amino acid transport and metabolism; H: Coenzyme transport and metabolism; M: Cell wall/membrane biogenesis; V: Defense mechanisms

^(2) ^GCV: GENECONV; MAX: Maximum χ^2^; NSS: Neighbour Similarity Score; PHI: Pairwise Homoplasy Test

^(3) ^ω = d_N_/d_S _(Number of nonsynonymous changes per nonsynonymous sites/Number of synonymous changes per synonymous sites); infinite values of ω (∞) indicate that the model did not find synonymous changes for the branches tested (d_S _= 0; ω ~ ∞). However, this (i.e., ω ~ ∞) does not affect the validity of the Likelihood Ratio Test, which was used to identify the genes under positive selection (Z. Yang, pers. Communication; see http://gsf.gc.ucdavis.edu/viewtopic.php?f=1&t=2079&sid=c4a82e1ca334ca84a00a8b85e0f33c9d and http://gsf.gc.ucdavis.edu/viewtopic.php?f=1&t=2329&sid=c4a82e1ca334ca84a00a8b85e0f33c9d;

^(4) ^Proportion of sites under positive selection

^(5) ^This column lists sites identified using Bayes Empirical Bayes (BEB) as being under positive selection; numbers identify the amino acid sites (in alignments) that are under positive selection

**Figure 3:**
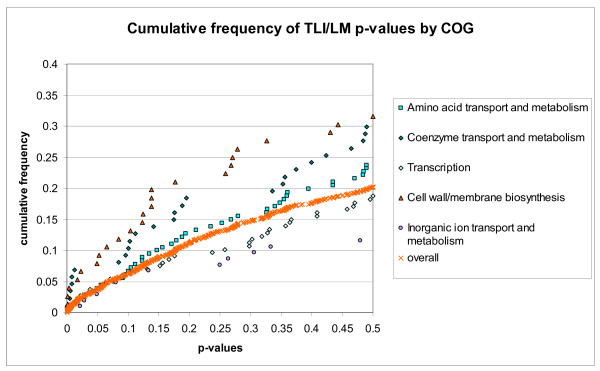
**Cumulative distribution of the *p*-values obtained from TLI/LM for selected COGs.** "Overall" represents all *p*-values regardless of the COG classification. Genes involved in "Cell-wall/membrane biosynthesis", "Coenzyme transport and metabolism", and "Amino acid transport and metabolism" showed a tendency to have lower *p*-values in comparison with all genes analyzed, while genes involved in "Transcription" and "Inorganic ion transport and metabolism" showed a tendency to have higher *p*-values than all genes analyzed.

While eight of the genes under positive selection (i.e., *lmo0098*, *lmo0653*, *lmo0782*. *lmo0785*, *lmo1424*, *lmo1529*, *lmo2215*, and *lmo2596*) encode membrane proteins [74], neither genes that encode for cell wall proteins nor genes that encode for secreted proteins or membrane proteins (based on the listings in LEGER) were significantly overrepresented among the 36 genes under positive selection (one-sided Fisher's exact test). One COG, "Signal transduction mechanisms", had a significant association with positive selection (nominal *P *= 0.008; one-sided Fisher's exact test) though, suggesting an enrichment for genes under positive selection in this category. However, after correction for multiple comparisons, the association is not significant (*P *= 0.098; Bonferroni correction). Because of the low number of genes under positive selection, it was not possible to assess the association between positive selection and most COGs. We thus assessed whether the distribution of the *p*-values for each test deviates from the random distribution for any of the COGs using a U-test. After Bonferroni correction, none of the COGs showed evidence for association with lower *p*-values (indicating evidence for positive selection) for either the lineage I (TLM1A) or the lineage II branch test (TLM2A). While three COGs (i.e., "Cell wall/membrane biogenesis", "Coenzyme transport and metabolism", and "Amino acid transport and metabolism") were associated with lower *p*-values for the TLI/LM test (see Fig. 3), only the association for the "Cell wall/membrane biogenesis" COG was significant after Bonferroni correction (nominal *P *= 0.004; Bonferroni corrected *P *= 0.036). Importantly, genes in this COG were not significantly associated with evidence for recombination, suggesting that a significant tendency for these genes to be under positive selection was not driven by an enrichment of genes with a history of recombination.

Among the 36 genes that showed evidence for positive selection, 29 genes also showed evidence for recombination, including five genes for which only one of the four recombination tests was significant. Statistical analyses showed that genes with evidence for recombination were overrepresented among the 36 genes found to be under positive selection (chi-square, *P *< 0.001). Among the seven genes with evidence for positive selection and no evidence for recombination, five and two genes showed evidence for positive selection in the lineage II ancestral branch and the *L. monocytogenes/L. innocua *branch, respectively; none of these genes showed evidence for positive selection in the lineage I ancestral branch.

### Core genome genes encoding MHC antigen do not show evidence for positive selection

The six core genome genes encoding antigens known to induce adaptive cellular immunity against *L. monocytogenes *in mice [75-82] were evaluated for evidence for positive selection. Epitopes in these antigens are presented to CD8^+ ^or CD4^+ ^T cells through MHC class Ia, Ib or II molecules. One MHC antigen (a putative 23 aa leader peptide encoded by a transcription attenuator upstream of *lmo2165*) showed no nonsynonymous changes and was thus not formally tested for positive selection. The other five MHC antigens (p60, encoded by *iap*; LemA, encoded by *lemA*; a lipoprotein encoded by *lmo1388*; an extracellular solute-binding protein encoded by *lmo0135*; and a protein with unknown function encoded by *lmo1602*) showed no evidence for positive selection. Moreover, the protein alignment of these five antigens showed no amino acid changes in the major epitope regions.

### Lineage II strains harbor more recombinant fragments than lineage I strains

**Figure 4:**
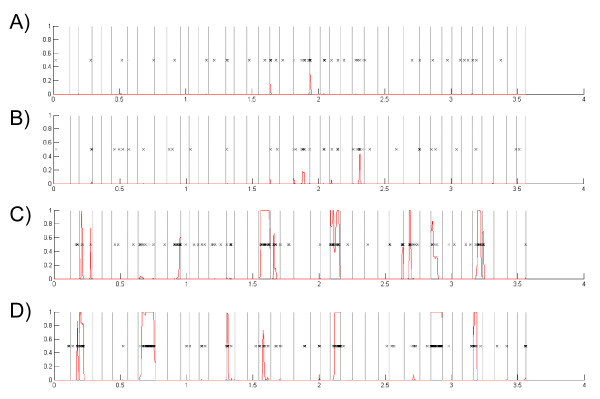
**Recombination events identified by Clonal Frame, using the concatenated alignment of 40 randomly selected genes, in the external branches of the *L. monocytogenes *strains (A) H7858, (B) F2365, (C) F6854, and (D) EGD-e. **Each of the 40 genes is represented between gray vertical lines. The order of the genes (left to right) is as follow: *clpX *(*lmo1268*), *lmo0343*, *lmo0405*, *pflC *(*lmo1407*), *phoP *(*lmo2501*), *lmo1436*, *lmo1460*, *lmo1537*, *hemC *(*lmo1556*), *ccpA *(*lmo1599*), *lmo1623*, *lmo1790*, *lmo2262*, *pepC *(*lmo2338*), *lmo2391*, *trxB *(*lmo2478*), *lmo0190*, *lmo0860*, *lmo0877*, *lmo1087*, *proA *(*lmo1259*), *lmo0992*, *smbA *(*lmo1313*), *lmo1401*, *lmo1420*, *opuCC *(*lmo1426*), *trpD *(*lmo1631*), *lmo1693*, *purK *(*lmo1774*), *lmo1825*, *panB *(*lmo1902*), *lmo0028*, *lmo2175*, *lmo2348*, *lmo2566*, *lmo0487*, *lmo0878*, *lmo1004*, *lmo1011*, *cbiH *(*lmo1199*). "x" indicate substitutions inferred to have occurred in the respective branches. Red lines represent the probability for each nucleotide to have been imported by means of recombination. Values at the bottom represent the position in the alignment in kilobases.

While the four recombination tests detailed above showed that a considerable number of genes in the *L. monocytogenes/L. innocua *core genome had evidence for recombination, these analyses did not allow for easy determination of the recipient strain and thus did not permit us to test the hypothesis that *L. monocytogenes *lineages differ in their frequency of gene fragment acquisition by recombination. ClonalFrame allows for identification of the recipient strains in recombination events and was thus used to analyze a set of 40 randomly selected genes (*clpX*, *lmo0343*, *lmo0405*, *pflC*, *phoP*, *lmo1436*, *lmo1460*, *lmo1537*, *hemC*, *ccpA*, *lmo1623*, *lmo1790*, *lmo2262*, *pepC*, *lmo2391*, *trxB*, *lmo0190*, *lmo0860*, *lmo0877*, *lmo1087*, *proA*, *lmo0992*, *smbA*, *lmo1401*, *lmo1420*, *opuCC*, *trpD*, *lmo1693*, *purK*, *lmo1825*, *panB*, *lmo0028*, *lmo2175*, *lmo2348*, *lmo2566*, *lmo0487*, *lmo0878*, *lmo1004*, *lmo1011*, and *cbiH*) for evidence of recombination and to determine the recipient strains in the recombination events that were identified. Due to computational constraints, testing larger number of genes was not easily feasible. The 40 genes were randomly chosen from a set of 1227 genes that showed no evidence for positive selection, had at least 5 informative sites and had alignment lengths between 600 and 1400 nucleotides. Among the 40 genes selected, 20 showed evidence for recombination in the genome-wide analysis. As recipient lineages cannot be reliably determined for recombination events in the lineage I and II ancestral branches, we only analyzed recombination events in the external branches (Fig. 4). Eleven recombination events were identified in the two lineage II strains (five in F6854 and six in EGD-e), while no recombination events were identified in the two lineage I strains (H7858 or F2365); in this data set, lineage I is thus significantly less likely to have recombination events as compared to lineage II (*P *< 0.001; Fisher's exact test).

### Analyses of five gene sequences obtained for 40 *L. monocytogenes *isolates confirm positive selection and recombination patterns observed in the genome-wide analyses

Five genes (Table 5) were selected for sequencing in a set of 40 *L. monocytogenes *isolates (representing lineage and source diversity; see Additional file 1) to confirm the positive selection and recombination patterns observed in the genome-wide analyses. The five genes chosen for these analyses included (i) *cheA *(*lmo0692*), which showed significant evidence for recombination (all tests significant) and positive selection with TLM1A; (ii) *lmo0693*, which showed evidence for positive selection with TLM2A and had no evidence for recombination; (iii) *flaR *(*lmo1412*), which showed evidence for positive selection with TLM2A and no evidence for recombination; (iv) *lmo2537*, which showed significant evidence for recombination with Maximum χ^2 ^but no evidence for positive selection; and (v) *phoP *(*lmo2501*), which showed evidence for recombination with GENECONV but showed no evidence for positive selection. Positive selection and recombination analyses were performed with gene alignments containing 45 sequences (40 gene sequences determined here as well as the respective gene sequences from the four *L. monocytogenes *and the one *L. innocua *genome [Table 1]).

**Table 5:** Positive selection and recombination analyses of 5 genes in 45 isolates^(1)^

Gene	Function	Recombination evidence^(2) ^(*p*-value)	Positive selection evidence^(3) ^(*p*-value)	ω^(4)^	p^(5)^	BEB^(6) ^sites (probability)
*cheA*	Two-component sensor histidine kinase CheA, involved in chemotaxis (Dons *et a*l., 2004)	GENECONV (< 0.001), NSS (< 0.001), Max χ^2 ^(< 0.001), PHI (< 0.001)	LIIIA/C-LI (< 0.001)	∞	0.002	140 (98%);
*lmo0693*	Putative flagellar motor switch protein, involved in motility	NSS (0.033)	LII (< 0.001)	∞	0.022	17 (73%) 18 (98%)
*flaR*	Histone like-DNA topology modulator, involved in regulation of flagellin expression (Sanchez-Campillo *et al*., 1995)	GENECONV (0.010), Max χ^2^(0.010), PHI (0.005)	LII (0.002)	14.1	0.046	4 (90%) 12 (99%) 68 (80%)
*phoP*	Putative two-component response phosphate regulator PhoP	GENECONV (0.014), NSS (0.001), Max χ^2 ^(0.020), PHI (0.003)	-	-	-	-
*lmo2537*	Putative UDP-N- acetylglucosamine-2-epimerase, involved in teichoic acid biogenesis (Dubail *et al*., 2006)	GENECONV (< 0.001), NSS (< 0.001), Max χ^2 ^(< 0.001), PHI (< 0.001)	-	-	-	-

^(1) ^Analyses were performed using an alignment of these 5 genes for the 40 *L. monocytogenes i*solates, for which these genes were sequenced here (see Supp. Table 1), as well as the four *L. monocytogenes *and one *L. innocua *strain for which full genome sequences were available (Table 1).

^(2) ^NSS: Neighbour Similarity Score; Max χ^2^: Maximum χ^2^; PHI: Pairwise Homoplasy Index; GENECONV performed with g-scale = 1 did not show significant inner fragments for any of the five genes, however four genes showed significant inner fragments in GENECONV performed with g-scale = 2, these *p*-values are reported here. The g-scale setting in GENECONV is associated with the number of polymorphisms allowed in a putative recombinant fragment; more polymorphisms are allowed as the g-scale value decreases from 3 to 1,

^(3) ^Branches where positive selection was identified. LIIIA/C-LI: ancestral branch of lineages IIIA/C and I isolates (branch B in Figure 5); LII: Ancestral branch of lineage II isolates (branch F in Figure 5); there was no evidence for positive selection in *phoP *and *lmo2537*.

^(4) ^ω = d_N_/d_S _(number of nonsynonymous changes per nonsynonymous sites/Number of synonymous changes per synonymous sites); infinite values of ω (∞) indicate that the model did not find synonymous changes for the branches tested (d_S _= 0; ω ~ ∞).

^(5) ^Proportion of sites under positive selection.

^(6) ^this column lists sites identified using Bayes Empirical Bayes (BEB) as being under positive selection; numbers identify the amino acid sites (in alignments) that are under positive selection; "probabilities" refer to the posterior probabilities that the respective sites evolved by positive selection.

Three genes (*lmo2537*, *phoP*, and *cheA*) showed significant evidence for recombination with all four recombination analyses (i.e., GENECONV, NSS, Maximum χ^2^, and PHI), consistent with the genome-wide analyses, which also found evidence for recombination in these genes. While *flaR *showed no evidence for recombination in the genome-wide analyses, analyses of the 45 *flaR *sequences showed significant evidence for recombination with Maximum χ^2^, PHI, and GENECONV (Table 5); these findings suggest that at least some of the additional *flaR *sequences represent recombinant alleles. Based on the 45 sequences, *lmo0693 *showed marginally significant evidence for recombination with NSS (P = 0.033) and no evidence for recombination with the other three methods, largely consistent with the genome-wide recombination analyses, which found no evidence for recombination in this gene.

ClonalFrame analysis on a concatenated alignment of the five genes (for all 45 isolates) identified 7, 2, 2, and 3 recombination events in the external branches for *cheA, phoP, flaR*, and *lmo2537*. No recombination events were identified in external branches for *lmo0693*. The recombination events identified in branches other than the ancestral branches leading to a given lineage involved 1, 7, 3, and 3 lineage I, II, IIIA/C, and IIIB branches as recipients in recombination events, respectively. Lineage II strains were significantly more likely to be involved as recipients in recombination events as compared to lineage I strains (*P *= 0.013, Fisher's exact test). ClonalFrame also allowed us to estimate the relative rate of recombination over mutation for these five genes. While, on average, mutations are 4.5 times more common than recombination events (95% IC = {3.0; 7.3}), a recombination event is 1.9 times more likely to change a single nucleotide than a point mutation (95% IC = {1.27; 2.6}).

**Figure 5:**
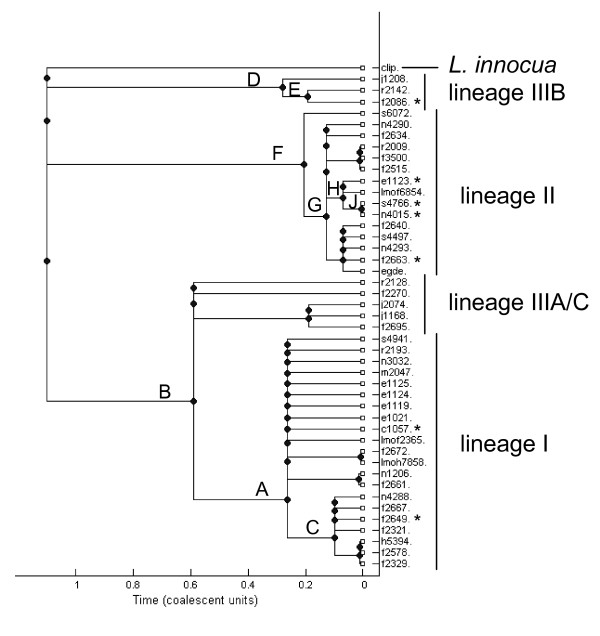
**Phylogenetic consensus tree generated by ClonalFrame from the concatenated alignment of *cheA*, *flaR*, *lmo0693*, *phoP *and *lmo2537 *for 45 isolates.** The 95% consensus phylogeny was obtained from two independent runs of ClonalFrame. This phylogeny clearly shows that the *L. monocytogenes *isolates form four distinct clusters, with lineage IIIA and IIIC (IIIA/C) isolates forming a sister group to lineage I isolates, while lineage IIIB isolates form an independent cluster that diverged earlier from the other isolates. Internal branches that showed evidence for recombination are labeled from A to J. Isolates with premature stop codons in *flaR *are marked with*.

Analyses of positive selection on the 45 sequences for the five genes yielded results similar to those obtained in the genome-wide analyses. As in the genome-wide analyses, we found no evidence for positive selection in the 45 sequences for *lmo2537 *and *phoP*. For *lmo0693 *and *flaR*, the lineage II ancestral branch was identified as evolving by positive selection (*P *< 0.001 and *P *= 0.002, respectively; the same branches were identified as being under positive selection in the genome-wide analyses). In *lmo0693*, two adjacent aa sites (17 and 18) were identified as being under positive selection, including one site identified with a posterior probability > 95% (Table 5). In *flaR*, three amino acid sites were identified as evolving by positive selection in the lineage II ancestral branch, including one site identified with a posterior probability > 95% (Table 5). While the genome-wide analysis for *cheA *found evidence for positive selection in the lineage I ancestral branch, analysis of the 45 sequences did not find evidence for positive selection in this branch (*P *= 0.207), but found significant evidence (*P *< 0.001) for positive selection in the ancestral branch that leads to lineages I and IIIA/C isolates (branch B, Fig. 5), a branch that was not present in the tree used in the genome-wide analyses (due to the absence of lineage IIIA/C strains in that data set). A single amino acid site was identified as evolving by positive selection in *cheA *(posterior probability > 95%; Table 5); isolates in lineage I and IIIA/C have glutamine at this site, while all other isolates, including *L. innocua *CLIP11262, bear an alanine. This amino acid site lies within the P2 domain of the CheA protein, which is the binding site for the response regulator, CheY; *cheY *itself seems to be extremely conserved (with no nonsynonymous change among the five strains included in the genome-wide analyses).

Interestingly, all three aa sites identified in *flaR*, *lmo0693*, and *cheA *as being under positive selection with posterior probabilities > 95%, involved nucleotide substitutions at all three codon positions (e.g., *cheA *aa site 140 GCC → CAG). These findings suggest that these codons evolved rapidly in the branches under positive selection. The nucleotide diversity in synonymous sites ranged from 0.141 to 0.151 among the three genes with evidence for positive selection while the nucleotide diversity in the synonymous sites of *lmo2537 *and *phoP*, which showed no significant evidence for positive selection, were 0.338 and 0.449, respectively. This suggests that significant tests for positive selection were not due to higher rates of synonymous substitutions, which could result in an underestimation of the number of synonymous changes due to recurrent mutations in synonymous sites.

### Unrelated *L. monocytogenes *isolates carry independently acquired premature stop codons in flaR

Among the 45 *L. monocytogenes flaR *sequences, we identified six sequences with distinct premature stop codons in *flaR *(Fig. 5), including five caused by frameshift mutations and one due to a nonsense mutation. The mutations leading to the premature stop codon were found in different regions of the sequence (Additional file 3). While one of these frameshift mutations was found in two isolates (FSL S4-766 and FSL N4-015, two closely related lineage II strains [Fig. 5]), all other mutations were only identified in one isolate each. *flaR *premature stop codons were found in different *L. monocytogenes *lineages, including lineage I (one frameshift and the nonsense mutation), lineage II (three different frameshift mutations), and lineage IIIB (one frameshift mutation) isolates. The isolates carrying premature stop codons in *flaR *were from human clinical cases (n = 4), animals (n = 2) and natural environment (n = 1). No statistical association could be identified between the presence of premature stop codon and source of the isolate (Fisher's exact test).

Since *flaR *has been previously shown to be involved in *L. monocytogenes *motility [83], swarming experiments were conducted to assess the swarming ability of six isolates presenting each of the six different mutations leading to premature stop codons, three isolates bearing full length *flaR *and an isogenic *flaR *null mutant. Isolates harboring naturally occurring premature stop codons in *flaR *showed, on average, significantly reduced swarming areas (*P *< 0.001; One-sided U-Test) and FSL F2-649, which harbor a premature stop codon in *flaR *due to a nonsense mutation, had the smallest swarming area among all natural isolates (*P *< 0.001; One-sided U-Test). Even though all isolates with *flaR *premature stop codons still showed some swarming and the 10403S Δ*flaR *showed no significant reduction in swarming ability, these findings suggest that the frameshift mutations in *flaR *may affect motility, at least in some strains.

## Discussion

We have chosen two closely related species within the genus *Listeria*, i.e., the pathogen *L. monocytogenes *and the non-pathogenic *L. innocua*, as a model system to further probe the evolution of bacterial pathogens using a comparative genomics approach. While comparative genomics approaches have provided important data on the importance of gene acquisitions in the evolution of bacterial pathogens in general [2,84,85] and in the evolution of *L. monocytogenes *in particular (e.g., by identifying a number of virulence genes associated with pathogenic *Listeria *spp.), our understanding of the contributions of recombination and positive selection to the evolution of the *L. monocytogenes/L. innocua *core genome has been limited so far.

### Recombination and positive selection both contribute to evolution of *L. monocytogenes*, but the relative contribution of these evolutionary forces differs among *L. monocytogenes *lineages

Overall, almost half of the genes present in *L. monocytogenes*/*L. innocua *core genome showed evidence for intragenic recombination (location of these genes is shown in Fig. 2). Only a much smaller proportion of the genes in the core genome (1.7%) showed evidence for positive selection. By comparison, a recent genomic study on the genus *Streptococcus *reported evidence for recombination in 18 to 37% of the genes in the core genome and evidence for positive selection in 11 to 34% of the genes in the core genome. This study was able to use a larger number of genome sequences (i.e., 26 genomes) and used different criteria for selection of genes with significant evidence for positive selection and recombination (i.e. only genes significant by all three tests for recombination were considered as recombinants) though [2]. While a comparison of the frequency of genes with evidence of recombination between *Listeria *and *Helicobacter pylori*, which appears to be panmictic [17], will be of interest, no genome wide studies of recombination in *Helicobacter *have been reported to date. In both gene-specific and multilocus sequence typing (MLST) studies, evidence has previously been found for contributions of recombination to the evolution of different *L. monocytogenes *genes, even though many of the genes identified as having an apparent history of recombination appear to be lineage and species specific, including a number of genes in the internalin family [4,9] and the *prfA *virulence gene cluster [12]. While some studies also have identified housekeeping genes with significant evidence for recombination [12], the magnitude of recombination in *Listeria *on a genome-wide level has not previously been known. Interestingly, it seems that although homologous recombination between closely related strains of *L. monocytogenes *is common, non-homologous recombination seems to be rare given the high synteny of the different *Listeria *genomes [[51], this study] and the relatively small number of strain and lineage specific genes (e.g., [83] and [51] genes specific to serotype 1/2a and 4b strains, respectively [51]). While others have also previously observed positive selection in *L. monocytogenes*, all previously identified genes under positive selection were virulence genes (i.e., *actA*, *inlA*, *inlB*, *inlC*, *inlC2 *and *inlF*) [4,9,12], which were specific to *L. monocytogenes *or selected *L. monocytogenes *lineages. We are thus the first to identify genes in the core genome which are under positive selection, indicating that positive selection does not just act on accessory and virulence genes.

Overall, most genes in the core genome found to be under positive selection also showed evidence for recombination; an association of recombination and positive selection was also previously observed in a genome-wide study on the evolution of genes in the genus *Streptococcus *[2]. Occurrence of both positive selection and recombination in specific genes has also previously been reported for selected *L. monocytogenes *virulence genes [4,9] as well as in other microorganisms [1,2,10,11,16].

While it has been shown that high recombination rates can lead to false positives in overall analysis using PAML, such as the TO test [86], no studies have evaluated the effect of recombination on the branch-site tests and it is unclear how recombination affects this analysis. The fact that, in our analyses, more than 1000 genes showed evidence for recombination but no evidence for positive selection suggests that the positively selected genes identified harbor distinct features that allowed their identification. As previously pointed out [2,9], some horizontally transferred fragments may also be more likely to be under positive selection, and positive selection may be important for fixation of recombinant gene and/or for adaptation of the newly acquired genes or alleles to a different function.

In analyses of sequence data for 40 genes, lineage II strains were significantly more likely to be identified as recipients of DNA fragments by horizontal gene transfer, indicating either more frequent lateral gene transfer or more frequent fixation of recombinant fragments. Higher frequency of recombinant fragments among lineage II strains has also previously been described for *L. monocytogenes *specific virulence genes (e.g., internalin genes [4,9]) and for some housekeeping genes [87]. The genetic basis of the promiscuity of lineage II isolates is currently unknown but it might be related to the observation that lineage II isolates appear to be overrepresented in foods, farms and natural environments [41], where *Listeria *bacteriophages (listeriophages) may be common due to the frequent presence of *L. monocytogenes *in some of these environments [88,89]. While listeriophages can perform generalized transduction [90], transduction between isolates of serotypes 1/2a and 4b has not been shown. Most listeriophages appear to be serotype-specific and phage host-specificity could account for the differences in recombination frequency between lineages. Alternatively, lineage I strains might have a more effective restriction systems for degradation of foreign DNA, a more efficient mismatch repair system that avoids incorporation of foreign DNA fragments into the chromosome, or may show reduced competency.

Our genome-wide analyses also indicated that positive selection occurred in more genes in the ancestral branch of lineage II as compared to the ancestral branch of lineage I. Interestingly, one gene with evidence for positive selection in the ancestral branch of lineage II encodes a putative transcriptional antiterminator of the BglG family (*lmo0297*). As another antiterminator of the BglG family (BvrA), which is only present in lineage II isolates [50], has been shown to be involved in cellobiose-dependent repression of PrfA-dependent virulence genes in *L. monocytogenes *[91], *lmo0297 *may have evolved to facilitate specific transcriptional repression functions in lineage II. Positive selection in the lineage II ancestral branch was further confirmed for two genes (*flaR*, *lmo0693*) in a larger isolate set. As lineage II strains are found in many different environments, including natural environment, farms, foods, animals with clinical disease, as well as human clinical cases (although less frequently than lineage I isolates) [41], one could hypothesize that lineage II isolates are exposed to a more diverse repertoire of distinct selective pressures than lineage I strains, which appear to be less common in natural environments and foods and seem to be more likely to be adapted to human or mammalian hosts [41,42].

### Diversification, by multiple mechanisms, of cell wall/membrane biogenesis and motility-related genes may play a particularly important role in the evolution of *L. monocytogenes*

In our study, genes involved in cell wall/membrane biogenesis showed a significant tendency to be identified as being under positive selection in the *L. monocytogenes*/*L. innocua *branch, suggesting that positive selection in these genes contributed to the divergence of these two species. *L. monocytogenes *genes encoding proteins involved in cell wall metabolism and encoding cell wall-anchored proteins have also previously been identified as harboring more nonsynonymous changes than genes involved in other functions [51], further supporting an important role for diversification of these gene categories in the evolution of *L. monocytogenes*. Specific surface associated proteins identified as being under positive selection included genes involved in transport of carbohydrates such as *mptD*, which appears to be involved in resistance to class IIa bacteriocins [92,93]. Class IIa bacteriocins are antimicrobial peptides frequently produced by lactic acid bacteria in foods [93], and resistance to these compounds are likely to confer an advantage to *L. monocytogenes *isolates in foods and environments. In addition, a putative glycosyl transferase (*lmo2121*) was identified as having evolved by positive selection in lineage I; glycosyl transferases could be associated with the differences in the somatic antigens in different serotypes and might be associated with strain differences in virulence and immunogenicity [51,94-96]. Functionally, active and rapid evolution of genes involved in cell wall/membrane biogenesis is likely to be important to allow bacteria to adapt to different and possibly rapidly changing environments, including, but not limited to, competing microorganisms as well as innate and adaptive immune system effectors in different host species. Our findings are consistent with a recent genome-wide study, which showed that most *E. coli *proteins that undergo positive selection are exposed on the cell surface, including a number of proteins known to interact with bacterial, host, or phage surface molecules [8].

A number of genes involved in flagellar synthesis and motility (e. g., *flaR*, *lmo0693 *and *cheA*) also showed evidence for positive selection, including when these genes were analyzed in a larger set of more diverse isolates. *lmo0693*, *cheA *and several other genes involved in motility and chemotaxis have also been found to be expressed more highly in four lineage I strains as compared to two lineage II strains [97], further suggesting a difference in regulation of motility and chemotaxis functions between the two lineages. Motility and flagellar expression appear to contribute to both biofilm formation [98] and host cells invasion [68,99,100] in *L. monocytogenes*, and diversification in these genes is likely to be important for adaptation to different host or non-host environments. Interestingly, seven isolates showed premature stop codons in *flaR*, including six due to frameshift mutations and one due to a nonsense mutation. While FlaR has been reported to be a histone-like protein that regulates transcription of the flagellin gene *flaA *in *L. monocytogenes *serotype 1/2c lineage II strain LO28 (as determined in a transposon mutant; [83]), a non-polar *flaR *null mutant in a serotype 1/2a lineage II strain (generated in our study reported here) did not show reduced motility at room temperature, even though the LO28 *flaR *mutant was reported to be non-motile at this temperature [83]. As shown here, natural isolates with premature stop codons in *flaR *showed, though, on average, significant reduced ability to swarm as compared to isolates harboring a full-length gene. *flaR *thus seems to have strain or perhaps serotype or lineage specific functions in *L. monocytogenes *and *flaR *inactivation in some strains is likely to be recent since most frameshift/nonsense mutations are isolate and not clade-specific. Alternatively, some or all frameshift and nonsense mutations could be reversible and *flaR *might be phase-variable, a tempting hypothesis as phase-variable flagella-related genes have been described in a number of other bacteria [101-104]. Interestingly, previous studies have shown that *inlA*, which encodes another *L. monocytogenes *surface protein that promotes mammalian host cell invasion, also carries several different premature stop codons in both lineage I and II strains [9,46,105-108]. The isolates with truncated InlA seem to be more common in foods [46] and show significantly reduced invasiveness for human intestinal epithelial cells [46], suggesting reduced virulence of these strains. Diversification by both positive selection and gene inactivation of genes encoding surface molecules with a role in virulence thus appears to be broadly important in the adaptation of *L. monocytogenes *to host and non-host associated environments.

## Conclusion

Our analyses reported here indicate that both recombination and positive selection contribute to the evolution of the *L. monocytogenes/L. innocua *core genome. While considerably more genes appear to be affected by recombination, positive selection still appears to play an important role in the evolution of both genes in the core genome (this study) as well as *L. monocytogenes *virulence genes that are not part of the core genome [4,9,12]. The list of genes identified as being under positive selection hopefully can be used by the scientific community to advance the discovery of genetic factors that allow this organism to adapt to diverse environments and hosts. In particular, our data suggest important roles for positive selection and diversification of genes encoding proteins associated with the cell wall and membrane biosynthesis on the evolution of *L. monocytogenes*.

Overall, genes in lineage I isolates were less likely to be affected by either recombination or positive selection, possibly reflecting that this lineage has experienced a recent bottleneck, as previously proposed [87]. Frequent recombination in combination with positive selection of some genes in lineage II strains, on the other hand, may be important for the evolution of this generalist lineage, which is present in many different environments and host, including human clinical cases (although less common than lineage I). In combination with previous studies that have shown considerable differences in frequency of recombination and positive selection among different *Streptococcus *lineages and species [2], our findings further show that even closely related bacterial lineages may differ in mechanisms contributing to their evolution.

## Abbreviations

COG: Clusters of Orthologous Groups of proteins; LRT: Likelihood Ratio Test; TO: Test Overall; an overall test for positive selection was carried out using the null model M1a (Nearly-neutral) and the alternative model M2a in PAML; TLM1A: Test *L. monocytogenes *lineage I Ancestral; this describes the branch-site test2 used to test for evidence of positive selection in the ancestral branch of *L. monocytogenes *lineage I.; TLM2A: Test *L. monocytogenes *lineage II Ancestral; this describes the branch-site test2 used to test for evidence of positive selection in the ancestral branch of *L. monocytogenes *lineage II; TLI/LM: Test *L. innocua*/*L. monocytogenes*; this describes the branch-site test2 used to test for evidence of positive selection in the branch separating *L. monocytogenes *and *L. innocua*.

## Authors' contributions

RHO outlined, performed, and interpreted the phylogenetic and statistical analyses, and drafted the manuscript. QS performed orthologous gene clustering and alignment, and implemented the analysis on the parallel computer cluster. MW supervised the project, participated in the design of the study and data interpretation, and finalized the manuscript. All authors read and approved the final manuscript.

## Supplementary Material

Additional file 1 Isolates used to confirm positive selection and recombination patterns in five selected genes. Click here for file

Additional file 2 Primers used for re-sequencing. Click here for file

Additional file 3 Alignment of the *flaR *sequence for seven isolates with premature stop codons. Click here for file
