# Supplementary material for: Genome-wide analyses reveal lineage specific contributions of positive selection and recombination to the evolution of Listeria monocytogenes
Source: BMC Evol Biol. 2008 Aug 12;8:233. doi: 10.1186/1471-2148-8-233 (PMC2532693; doi:10.1186/1471-2148-8-233)
Supplement: Additional file 1 — Isolates used to confirm positive selection and recombination patterns in five selected genes. [file 1471-2148-8-233-S1.doc]

**Supplemental Table 1.** Isolates used to confirm positive selection and recombination patterns in five selected genes.

| **Isolate** | **Serotype** | **Source** | **Source, specific** | **Year** | **State** | **Lineage** |
| --- | --- | --- | --- | --- | --- | --- |
| FSL E1-119 | 1/2b | animal | bovine | 2000 | NY | I |
| FSL F2-329 | 1/2b | animal | sheep | 2000 | NY | I |
| FSL F2-578 | 1/2b | food | finished RTE^(1)^ food product (Meat) | 2000 | NY | I |
| FSL F2-649 | 1/2b | human | human sporadic | 2001 | OH | I |
| FSL F2-667 | 1/2b | food | finished RTE food product (Meat) | 2001 | NY | I |
| FSL H5-394 | 1/2b | animal | bovine | 2004 | NY | I |
| FSL N4-288 | 1/2b | animal | clinical | 2001 | NY | I |
| FSL C1-057 | 4b | human | human sporadic | 1998 | NY | I |
| FSL E1-021 | 4b | animal | avian | 1998 | NA | I |
| FSL E1-124 | 4b | animal | bovine | 2001 | NY | I |
| FSL E1-125 | 4b | animal | caprine | 2000 | NY | I |
| FSL F2-321 | 4b | food | lunch meat | 2000 | CA | I |
| FSL F2-661 | 4b | human | human sporadic | 2001 | NY | I |
| FSL F2-672 | 4b | human | human sporadic | 2001 | NY | I |
| FSL M2-047 | 4b | human | human epidemic | 1999 | NY | I |
| FSL N1-206 | 4b | human | human epidemic | 1998 | OH | I |
| FSL N3-032 | 4b | animal | caprine | 2001 | NY | I |
| FSL R2-193 | 4b | food | smoked seafood | 2001 | MD | I |
| FSL S4-941 | 4b | non-food environment | vegetation | 2002 | NY | I |
| FSL E1-123 | 1/2a | animal | bovine | 2001 | NY | II |
| FSL F2-515 | 1/2a | food | finished RTE food product (Meat) | 2000 | NY | II |
| FSL f2-634 | 1/2a | human | human sporadic | 2001 | NY | II |
| FSL F2-663 | 1/2a | human | human sporadic | 2001 | NY | II |
| FSL F3-500 | 1/2a | human | human sporadic | 2003 | NY | II |
| FSL N4-015 | 1/2a | animal | bovine | 2002 | NY | II |
| FSL N4-290 | 1/2a | animal | clinical | 2001 | NY | II |
| FSL N4-293 | 1/2a | animal | clinical | 2001 | NY | II |
| FSL R2-009 | 1/2a | food | deli salad | 2000 | MD | II |
| FSL S4-497 | 1/2a | non-food environment | soil | 2002 | NY | II |
| FSL S4-766 | 1/2a | non-food environment | vegetation | 2002 | NY | II |
| FSL S6-072 | 1/2a or 3a | non-food environment | water | 2002 | NY | II |
| FSL F2-640 | 1/2c | food | raw meat/poultry | 2001 | NY | II |
| FSL F2-086 | 4a | human | human sporadic | 1999 | NY | III |
| FSL F2-270 | 4a | human | human sporadic | 1999 | OH | III |
| FSL F2-695 | 4a | human | human sporadic | 2001 | NY | III |
| FSL J1-168 | 4a | human | human sporadic | 1996 | NY | III |
| FSL J1-208 | 4a | animal | caprine | 1998 | GA | III |
| FSL R2-128 | 4a | food | bagged salad | 2001 | MD | III |
| FSL R2-142 | 4a | food | bagged salad | 2001 | MD | III |
| FSL J2-074 | 4c | animal | bovine | 1993 | OH | III |

^(1)^ RTE: ready-to-eat
