# Supplementary material for: Genome-wide analyses reveal lineage specific contributions of positive selection and recombination to the evolution of Listeria monocytogenes
Source: BMC Evol Biol. 2008 Aug 12;8:233. doi: 10.1186/1471-2148-8-233 (PMC2532693; doi:10.1186/1471-2148-8-233)
Supplement: Additional file 2 — Primers used for re-sequencing. [file 1471-2148-8-233-S2.doc]

|  | Primer | | Sequence 5’-3’ | PCR Annealing T ºC | PCR/Seq |
| --- | --- | --- | --- | --- | --- |
| *cheA* | |  |  |  |  |
|  | cheAF | | CAAGGTATGGTTGTAGACGCC | 50ºC | PCR/ Seq |
|  | cheAR | | CTTTCGGTTCCCATTCAGG | 50ºC | PCR/ Seq |
|  | cheAF2 | | GCCAGACAGAGAAGCGATTG | - | Seq |
|  | cheAR2 | | GCGCCGACTCTAGGACTACTTC | - | Seq |
|  | cheAF3 | | CCACAATTAATCTAAAAGCTTTCCATAG | - | Seq |
|  | cheAR3 | | CTAACACTTTGCGTTTATTGATTCC | - | Seq |
|  | |  |  |  |  |
| *phoP* | |  |  |  |  |
|  | EGDphoPF | | GATTAATATAAATGCATTTCTACCCG | 55 ºC →45 ºC | PCR/ Seq |
|  | EGDphoPR | | GTACGTTGATTTCATTAGTTCCCC | 55 ºC →45 ºC | PCR/ Seq |
|  |  | |  |  |  |
| *flaR* | |  |  |  |  |
|  | Lmo1412F | | TATCCAAAATAAAAACTGGTAAAGTAAG | 50ºC | PCR/ Seq |
|  | Lmo1412R | | ATCGCAAACAACCAATAAACAC | 50ºC | PCR/ Seq |
|  | |  |  |  |  |
| *lmo2537* | |  |  |  |  |
|  | Lmo2537F | | GTCCAGGCTTAGGAGATGCTG | 53ºC | PCR/ Seq |
|  | Lmo2537R | | CGGCGGGATTGTAGTGAATAC | 53ºC | PCR/ Seq |
|  | Lmo2537F2 | | GTTTAATACTTATGACGGCTCACC | - | Seq |
|  | Lmo2537R2 | | CACGACTTTCCACGATTTCTC | - | Seq |
|  | |  |  |  |  |
| *lmo0693* | |  |  |  |  |
|  | Lmo693F | | GCAACGATTTTAGGCGATGG | 54ºC | PCR/ Seq |
|  | Lmo693R | | TCTCTTGGTCCAATTTCATCTTCAC | 54ºC | PCR/ Seq |

**Supplemental Table 2.** Primers used for re-sequencing.
