# Supplementary material for: Genome-wide analyses reveal lineage specific contributions of positive selection and recombination to the evolution of Listeria monocytogenes
Source: BMC Evol Biol. 2008 Aug 12;8:233. doi: 10.1186/1471-2148-8-233 (PMC2532693; doi:10.1186/1471-2148-8-233)
Supplement: Additional file 3 — Alignment of the flaR sequence for seven isolates with premature stop codons. [file 1471-2148-8-233-S3.doc]

FSL-C1057 ATG-37N-AGAAAAA-GTAT**TAA**TAATTGG-81N-TTTTTGGCTG-42N-GTGA-ACGGAATTCTGCGTTCTGAAAAAAA-GTATATTATAGATGGTGATTA-296N

FSL-E1123 ATG-37N-AAGAAAAAGTGTTGATAATCGG-81N-TTTCTGGCAG-42N-GTGG-ACAACATTATGAGTTCTAAAAAAAAAGTATATTATAGATGG**TGA**TTA-296N

FSL-F2086 ATG-37N-AGAAAAAAGTGTTAATAATCGG-81N-TTTTTGGCTG-42N-GTAA-ACAGAATTCTGCTTTCTGAAAAAA--GTATATTA**TAG**ATGGTGATTA-296N

FSL-F2649 ATG-37N-AGAAAAAAGTATTAATAATTGG-81N-TTTT**TAG**CTG-42N-GTGA-ACGGAATTCTGCGTTCTGAAAAAAA-GTATATTATAGATGGTGATTA-296N

FSL-F2663 ATG-37N-AAGAAAAAGTGTTGATAATCGG-81N-TTTCTGGCAG-42N-GTGG-ACAACATTATGAGTTCTAAAAAAA--GTATATTA**TAG**ATGGTGATTA-296N

FSL-N4015 ATG-37N-AAGAAAAAGTGTTGATAATCGG-81N-TTTCTGGCAG-42N-GTGGGACAACATTATGAGTTC**TAA**AAAAA--GTATATTATAGATGGTGATTA-296N

FSL-S4766 ATG-37N-AAGAAAAAGTGTTGATAATCGG-81N-TTTCTGGCAG-42N-GTGGGACAACATTATGAGTTC**TAA**AAAAA--GTATATTATAGATGGTGATTA-296N

F2365 ATG-37N-AGAAAAAAGTATTAATAATTGG-81N-TTTTTGGCTG-42N-GTGA-ACGGAATTCTGCGTTCTGAAAAAAA-GTATATTATAGATGGTGATTA-296N

CLIP ATG-37N-AGAAAAAAGTGTTAATAATTGG-81N-TTTTTGGTTG-42N-GTGA-ACGGAATTCTGCGTTCTGAAAAAAA-GTATATTATAGATGGTGATTA-296N

EGD-e ATG-37N-AAGAAAAAGTGTTGATAATCGG-81N-TTTCTGGCAG-42N-GTGG-ACAACATTATGAGTTCTAAAAAAAA-GTATATTATAGATGGTGATTA-296N

**Supplemental Figure 1.** Alignment of the *flaR* sequence for seven isolates with premature stop codons. Sequences for EGD-e, F2365 and CLIP 11262 (CLIP) were included for comparison. Indels resulting in frameshift are highlighted in gray. Premature stop codons are bolded and underlined. Note that the FSL F2-649 premature stop codon is due to a nonsense mutation and not a frameshift mutation.
